# An Exosomal miRNA Biomarker for the Detection of Pancreatic Ductal Adenocarcinoma

**DOI:** 10.3390/bios12100831

**Published:** 2022-10-06

**Authors:** Amy Makler, Ramaswamy Narayanan, Waseem Asghar

**Affiliations:** 1 Micro and Nanotechnology in Medicine, College of Engineering and Computer Science, Boca Raton, FL 33431, USA; 2 Department of Biomedical Science, Charles E. Schmidt College of Medicine, Florida Atlantic University, Boca Raton, FL 33431, USA; 3 Department of Biological Sciences, Florida Atlantic University, Boca Raton, FL 33431, USA; 4 Department of Biology, University of North Florida, Jacksonville, FL 32224, USA; 5 Department of Computer & Electrical Engineering and Computer Science, Florida Atlantic University, Boca Raton, FL 33431, USA

**Keywords:** biomarker, cancer, diagnostics, exosomes, miRNA, pancreatic cancer

## Abstract

Pancreatic ductal adenocarcinoma (PDAC) remains a difficult tumor to diagnose and treat. To date, PDAC lacks routine screening with no markers available for early detection. Exosomes are 40–150 nm-sized extracellular vesicles that contain DNA, RNA, and proteins. These exosomes are released by all cell types into circulation and thus can be harvested from patient body fluids, thereby facilitating a non-invasive method for PDAC detection. A bioinformatics analysis was conducted utilizing publicly available miRNA pancreatic cancer expression and genome databases. Through this analysis, we identified 18 miRNA with strong potential for PDAC detection. From this analysis, 10 (MIR31, MIR93, MIR133A1, MIR210, MIR330, MIR339, MIR425, MIR429, MIR1208, and MIR3620) were chosen due to high copy number variation as well as their potential to differentiate patients with chronic pancreatitis, neoplasms, and PDAC. These 10 were examined for their mature miRNA expression patterns, giving rise to 18 mature miRs for further analysis. Exosomal RNA from cell culture media was analyzed via RTqPCR and seven mature miRs exhibited statistical significance (miR-31-5p, miR-31-3p, miR-210-3p, miR-339-5p, miR-425-5p, miR-425-3p, and miR-429). These identified biomarkers can potentially be used for early detection of PDAC.

## 1. Introduction

Pancreatic ductal adenocarcinoma (PDAC) remains one of the most dismal types of cancers diagnosed with a 5-year survival rate of 10% [1]. PDAC is projected to overtake both breast and colorectal cancers as the second leading cause of cancer-related deaths before 2030 [2,3]. PDAC is typically asymptomatic and only 10–25% of patients are diagnosed in the early stages, while the majority are diagnosed during late stage disease [4]. Additionally, 60–80% of patients presenting resectable pancreatic tumors will exhibit recurrence, regardless of administration of adjuvant therapy [5,6]. To date, the only protein marker approved and designated for PDAC diagnosis and monitoring is Cancer Antigen 19-9 (CA19-9). CA19-9 is released at low levels by organs of the gastrointestinal tract as well as endometrial cells [7]. Elevated levels of the protein may be present in PDAC as well as various diseases including pancreatitis and gastrointestinal disorders and tumors [7,8]. Additionally, CA 19-9 is recommended as a prognostic indicator and not suggested for screening or as an early detection marker for PDAC [9,10]. Hence, there is a dire need for alternative screening methods in which high-risk patients may be monitored for the potential of not only developing PDAC but also monitoring for the presence of resurgent tumors.

Patients with high risk of developing PDAC are those with a history of smoking, obesity, chronic pancreatitis, hereditary PDAC, and onset of diabetes after 50 years of age [11]. However, patients who smoke and exhibit chronic pancreatitis are the most at-risk for PDAC development [12]. It has been estimated that smoking increases the risk of PDAC by nearly 6x while chronic pancreatitis increases susceptibility by nearly 8-fold [12,13]. Additionally, pancreatic cysts are associated with PDAC risk, particularly if a patient presents with multiple lesions [14]. Pancreatic cysts are precancerous lesions such as pancreatic intraepithelial neoplasms (PanINs), intraductal papillary mucinous neoplasms (IPMNs), and mucinous cystic neoplasms (MCNs). PanINs are the most common types of precancerous lesions, oftentimes leading to invasive carcinoma of the pancreatic ducts. IPMNs are less common while MCNs are the least frequent [15,16]. Like PDAC, chronic pancreatitis and pancreatic lesions are difficult to detect as well as diagnose and require a combination of blood tests, imaging scans, and invasive biopsies [17,18]. Patients who are diagnosed and monitored do exhibit an overall decrease in PDAC risk [12,13]. Thus, early detection and monitoring are imperative to ensure overall positive outcomes for patients.

Since monitoring high-risk patients has proven beneficial for long-term prevention of PDAC, it is necessary to detect PDAC early in the event a patient’s condition does not improve. Unfortunately, early detection of PDAC remains elusive and current methods of detection are not sufficient for early diagnosis. Non-invasive liquid biopsy methods of diagnosis are attractive for the detection of solid tumors as they avoid the need for invasive surgeries. One such non-invasive method is to utilize secreted exosomes for cancer diagnosis and prognosis. Exosomes are 40–100 nm-sized extracellular vesicles with a lipid bilayer membrane and contain DNA, RNA, and protein cargos [19]. Exosomes are secreted in diverse body fluids, thus making them ideal for blood or urine-based diagnostic biomarkers discovery [20]. Studies have shown that exosome number alone may be indicative of tumor presence and burden [21,22,23,24]. Additionally, exosomal cargo can exhibit specificity for certain tumors and this has been observed for breast [25], lung [26], prostate [27], and pancreatic cancers [28,29,30]. Exosomes have also been utilized for early tumor detection, even before the onset of clinical evidence [31]. Thus, it is possible to use exosomes and exosomal contents to monitor high-risk patients.

Noncoding RNAs (ncRNAs) play key roles in development, gene regulation, and in the etiology of various diseases [32,33]. There are several subtypes of ncRNAs, including long noncoding RNA (lncRNA), microRNA (miRNA), and piwi interacting RNA (piRNA). Their regulatory functions are diverse and vary depending on the subcategory, but they frequently engage in epigenetic modifications as well as protein silencing and degradation as part of normal regulatory functions [34]. Thus, the deregulation of these ncRNAs have been heavily implicated in both the onset and the progression of tumors [35,36,37]. Additionally, many of these deregulated ncRNAs can be found in the cytoplasm where they are packaged into exosomes to be utilized for the diagnosis of various tumors. Because of this, exosomal ncRNAs are being investigated as biomarkers for various cancers [24].

We conducted an extensive bioinformatics screening of publicly available cancer ncRNA expression databases to identify candidate exosomal ncRNAs associated with PDAC. Our extensive analysis yielded 10 exosomal miRNA transcripts as a potential biomarker for PDAC. We used RT-qPCR to enrich the differentially expressed mature miRNAs extracted from exosomes obtained from three distinct PDAC cell culture models and one control model from an immortalized pancreatic duct epithelial cell culture. Seven mature miRNAs (miR-31-5p, miR-31-3p, miR-210-3p, miR-339-5p, miR-425-5p, miR-425-3p, and miR-429) were found to be differentially expressed between at least one PDAC cell culture model compared to the control immortalized pancreatic duct epithelial cell culture model. These seven exosome-derived miRNAs could serve as a novel non-invasive diagnostic panel for PDAC.

## 2. Materials and Methods

### 2.1. Database Generation and Biomarkers Identification

A database of pancreatic cancer-associated noncoding RNAs was generated using the following meta knowledgebases: Disease Gene Network [38], GeneCards [39], and NCBI gene. Expression databases including the pancreatic cancer database [40], miR2disease [41], miRCancer [42], and lnc2cancer [43] were accessed to facilitate the development of this PDAC-specific exosomal ncRNA database. The following keywords and search terms were utilized to extract PDAC-associated genes from GeneCards, NCBI Gene: (pancreatic cancer, pancreas cancer, pancreatic neoplasm, pancreatic adenocarcinoma, acinar cell carcinomas or adenosquamous carcinomas, squamous cell carcinomas, signet ring cell carcinomas, undifferentiated carcinomas, carcinoma of the ampulla of Vater, islet cell tumors, Gastrinomas, Insulinomas, Glucagonomas, Somatostatinomas, VIPomas, pancreatic polypeptide cancer, Serous cystic neoplasms, Mucinous cystic neoplasms, Intraductal papillary mucinous neoplasms, Solid pseudopapillary neoplasm, and acinar cell carcinoma). The ncRNA database was enriched for association and secreted nature based off previous work using the GeneCards suite [39] containing GeneALaCart, GeneAnalytics, and VarElect as well as the Exosome Encyclopedia, ExoCarta [44], and gene ontology tool QuickGO [20,45]. The HUGO Gene Nomenclature Committee was used to validate the ncRNA symbols present in the database. Exosomal ncRNA were then analyzed for copy number alteration using the PDAC UTSW dataset from the cBioPortal [46] tool, with the goal of determining which ncRNAs exhibited alteration in at least 10% of the patients sampled. Additional parameters included determining which ncRNAs exhibited deregulation (either overexpression or underexpression) and differential expression in chronic pancreatitis patients, precancerous lesions, and PDAC using the pancreatic expression database (PED) [47]. Biomarkers and their expression in PDAC, precancerous lesions, pancreatitis, and cell lines were partially verified by the pancreatic cancer database [40].

### 2.2. Cell Lines

All cell lines were purchased from the American Type Culture Collection in 2019. Cells were used within 8 months of purchase for analysis; cell lines were not re-authenticated nor was mycoplasma testing conducted. PDAC cell lines PANC1 (ATCC^®^ CRL-1469), BXPC3 (ATCC^®^ CRL-1687), and CAPAN2 (ATCC^®^ HTB-80) were chosen for their differences in mutation profile. PANC1 has mutations in KRAS and p53, BXPC3 possesses mutant p53 and wildtype KRAS, and CAPAN2 exhibits a KRAS mutation and is wildtype for p53. We used pancreatic duct epithelial cell line hTERT-HPNE E6/E7/st (ATCC^®^ CRL-4037) as a non-cancerous control cell line. This is a stable cell line immortalized by telomerase catalytic subunit, with p53 and Rb deactivation by HPV oncogenes E6 and E7, respectively. SV40 large T protein is a simian-derived oncogene which was introduced to this cell line by Campbell et al. in 2007 to sensitize the cell line to KRAS mutation [48]. All cell lines were cultured as per ATCC guidelines specific for each cell line; however, standard FBS was replaced with exosome depleted FBS as media supplement for all cell lines. PANC1 media is comprised of 450 mL of DMEM, 50 mL of exosome-depleted FBS, and 5 mL of penicillin-streptomycin; BCPC3 media includes 450 mL RPMI-1640, 50 mL of exosome-depleted FBS, and 5 mL of penicillin-streptomycin; CAPAN2 media was formulated using 450 mL of McCoy’s 5a Medium, 50 mL exosome-depleted FBS, and 5 mL of penicillin-streptomycin; and hTERT-HPNE E6/E7/st base medium is comprised of 375 mL of low glucose DMEM (Sigma Aldritch Cat# D-5030), 125 mL Medium M3 Base (Incell corporation, cat# M300F500), 27 mL exosome-depleted FBS, 5.4 mL of hr EGF stock (1 µg/mL, Gibco cat# PHG0314), 5.4 mL L-glutamine (ATCC cat# 30-2214), 1.4 mL D-glucose (Sigma cat# G8644), and 5 mL of penicillin-streptomycin. To prepare the 1 µg/mL of EGF stock solution, 1 vial of 10 µg EGF is combined with 10 mL of PBS and 10% BSA and filter sterilized before adding the EGF.

### 2.3. Exosome Isolation from Cell Culture Media

Cell cultures were grown for two passages before they were split into three T25 flasks and cultured for three days in 5 mL of their respective media. All cell lines exhibited at least 80% confluency and 90% viability as determined by Trypan Blue staining. Volumes of cell culture media from each sample were collected in accordance to a normalized cell count of 1 × 10^6^ cells/mL across all cell lines and biological replicates. Exosomes were isolated from each normalized sample using the Thermofisher^®^ Total Exosome Isolation Kit (for cell culture media) as per manufacturer directions.

### 2.4. Isolation and Profiling of Exosomal miRNA

After completion of exosome isolation, exosomal miRNAs were extracted using the Thermofisher^®^ Total RNA and Protein Isolation kit as per manufacturer instructions. Before the phenol-chloroform extraction step, a spike-in of 1.5 pg of cel-miR-2-3p (Thermofisher) was added to the solution containing the exosomes to monitor RNA extraction efficiency and provide an exogenous control for RTqPCR. Additionally, an endogenous control miR-16-5p was used. The Thermofisher^®^ Taqman™ Advanced cDNA synthesis kit and the Thermofisher^®^ Taqman™ Advanced miRNA assay probes for the following miRs was used to measure exosomal miRNA expression: cel-miR-2-3p (Assay ID: 478291_mir), miR-16-5p (Assay ID: 477860_mir), miR-31-3p (Assay ID: 478012_mir), miR-31-5p (Assay ID: 478015_mir), miR-93-3p (Assay ID: 478209_mir), miR-93-5p (Assay ID: 478210_mir), miR-133a-3p (Assay ID: 478511_mir), miR-133a-5p (Assay ID: 478706_mir), miR-210-3p (Assay ID: 477970_mir), miR-210-5p (Assay ID: 478765_mir), miR-330-3p (Assay ID: 478030_mir), miR-330-5p (Assay ID: 478830_mir), miR-339-3p (Assay ID: 478325_mir), miR-339-5p (Assay ID: 478040_mir), miR-425-3p (Assay ID: 478093_mir) miR-425-5p (Assay ID: 478094_mir), miR-429 (Assay ID: 477849_mir), miR-1208 (Assay ID: 478637_mir), miR-3620-3p (Assay ID: 479690_mir), and miR-3620-5p (Assay ID: 480850_mir). The advanced Taqman™ system for miRNA detection uses FAM for the reporter dye and ROX for standard. The AriaMX thermocycler was used for RTqPCR. Cq values of miR-16-5p and cel-mir-2-3p were averaged to provide a stable control value. The relative expression levels of all miRNAs across all cell lines were calculated using ΔΔCq, with a threshold value of 0.1, and outliers removed. The cell line hTERT-HPNE E6/E7/st was used as the control cell line. Student’s *t*-test was conducted to determine statistically significant differences in relative expression levels of miRNA.

Standard qPCR values consider >35 to be background noise. However, in qPCR of miRNA, if a value is >35 or does not exhibit a Cq value, it is considered to exhibit an unreliably low detection. Regardless, this information provides important data that would otherwise be missed if those numbers were ignored. To compensate and ensure the observation of significant differences in the expression of miRs across the cell lines, all values of ≥35 or undetermined values were replaced with an arbitrary low value of 36. Additionally, miRs with Cq ≤ 35 in <20% of the samples were excluded as were samples which did not exhibit any values, as previously described [49].

### 2.5. microRNA Nomenclature

MicroRNA have different naming schemes, depending on their level of processing. MIR refers to the gene encoding the miRNA; mir designates the pre- and primary transcript; miR denotes the mature form of the miRNA after it has been processed [50].

## 3. Results

### 3.1. Establishment of a Noncoding RNA Biomarker

#### 3.1.1. Database Generation and Lead Identification

**Figure 1:**
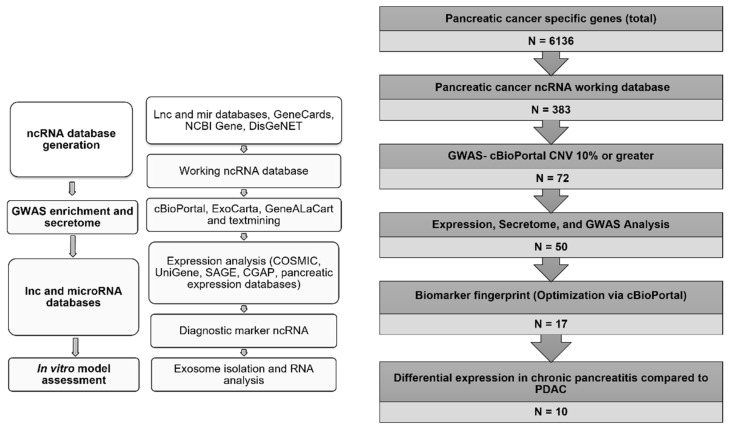
Workflow for the identification and analysis of exosomal microRNA for PDAC diagnosis. Whole gene lists associated with PDAC were downloaded from the lnc and miRNA databases as well as GeneCards, NCBI Gene, and DisGenNET in order to identify as many PDAC-associated ncRNAs as possible (N = 6136). Protein-coding genes were then removed in order to isolate only ncRNAs (N = 383). This provided the basis for our ncRNA database. cBioPortal was used to identify genetic alterations of ncRNAs across patient data in order to determine the most attractive targets for diagnostic potential (N = 72). The expression databases (COSMIC, UniGene, SAGE, CGAP, and PED) and secretome tools (ExoCarta and GeneALaCart) were then accessed to determine if any of the remaining MIRs were secreted and exhibited changes in expression in PDAC to better determine the most reliable exosomal targets for PDAC detection (N = 50). Additional optimization using cBioPortal with putative secreted exosomal MIR markers (CNV 15% or greater) was used (N = 18). Comparison of MIR expression in chronic pancreatitis compared to PDAC was then used as the final metric for candidate MIRs for diagnostic potential (N = 10). The MIRs identified in this manner were considered diagnostic markers for further analysis in cell culture models.

Our workflow strategy incorporates extensive bioinformatics analysis to identify potential leads for in vitro analysis (Figure 1). In order to generate this database, multiple knowledgebases were accessed to determine associated PDAC genes (N = 6136). Upon analysis and enrichment of the datasets provided by these various knowledgebases, a database of 383 PDAC-associated ncRNA was generated (Supplementary File S1). The majority (86%) of the ncRNAs in the generated database belong to the MIR subtype (Figure 1). This provided the basis for a panel of miRNA for PDAC detection. To generate a dataset of MIRs which exhibited alteration in PDAC patients, the cBioPortal tool was utilized. The cBioPortal tool provides valuable information regarding copy number alteration for a given gene of interest as well as tumor staging. It was predicted that if enough individuals exhibited alterations in the MIRs of interest it would translate as a change of expression of those MIRs in affected individuals. This would then potentially provide a fingerprint of potential miRNAs for PDAC detection. The University of Texas South Western (UTSW) PDAC dataset (N = 109) was used to batch analyze the 383 ncRNAs in the database. To be considered for further analysis, the cBioPortal tool had to report a copy number alteration of the specific ncRNA in a minimum of 10% of the patients in the sample. This criterion yielded 72 miRNAs.

These 72 MIRs were enriched for their presence in exosomes using the exosome encyclopedia tool, ExoCarta, which provides general evidence for the presence of miRs in exosomes. This analysis yielded 50 exosomal MIRs (Supplementary File S2). We then used the cBioPortal batch analysis function on the UTSW dataset to test combinations of exosomal MIRs enriched from our dataset to determine the most optimal MIRs to collectively test as a potential diagnostic panel. The final panel comprised 18 MIRs (MIR27A, MIR31, MIR93, MIR96, MIR122, MIR130B, MIR133A1, MIR203A, MIR210, MIR330, MIR339, MIR425, MIR429, MIR522, MIR590, MIR664A, MIR1208, and MIR3620), Altogether, 90% of the patients in the UTSW dataset from cBioPortal exhibited alterations in at least one of the 18 MIRs proposed for potential PDAC detection.

A comprehensive analysis yielded a panel of 18 exosomal MIRs associated with PDAC. Collectively, these 18 MIRs exhibited alteration in 90% of the UTSW patient dataset (N = 109) from cBioPortal. These MIRs provide the basis for the design of a diagnostic panel with the potential for early detection and monitoring of PDAC.

#### 3.1.2. Selection of Biomarker Panel for Diagnostics and Screening

Tumor progression is marked by chronic inflammation and resultant aberrant gene expression. It was therefore of interest to establish whether the miRNAs in the proposed panel exhibited progressive deregulation associated with chronic pancreatitis and tumor stage progression. Expression of the miRNAs in chronic pancreatitis and PDAC were predicted using the pancreatic expression database (PED) [47] and partially verified using the pancreatic cancer database (PCD) [40] (Supplementary File S3). The presence of altered miRNAs in proceeding PDAC stages was predicted using the Staging clinical track provided by cBioPortal. These tools provided additional optimization of the proposed diagnostic and screening panel.

Using the cBioPortal clinical track for staging and UTSW dataset, seven patients were diagnosed with stage I PDAC. These seven patients exhibited amplifications or deletions (“alterations”) in 13 of the miRNAs (MIR27A, MIR31, MIR93, MIR96, MIR122, MIR130B, MIR203a, MIR210, MIR330, MIR339, MIR425, MIR429, and MIR3620). Most patients in the dataset (N = 94) displayed stage II PDAC and all 18 MIRs exhibited alteration in these patients. Eleven miRNAs (MIR93, MIR96, MIR31, MIR130B, MIR133A1, MIR210, MIR330, MIR429, MIR522, MIR590, and MIR1208) manifested alterations in stage III patients (N = 6). Only two patients in the dataset were diagnosed with stage IV PDAC. Six miRNAs (MIR27A, MIR203A, MIR210, MIR429, MIR664A, and MIR1208) were altered in these stage IV patients (Table 1). Additionally, four miRNAs (MIR27A, MIR203A, MIR210, MIR429) were shared between stages I and IV. Meanwhile stages III and IV appeared to share three miRNAs (MIR210, MIR429, and MIR1208).

Because chronic pancreatitis increases the risk of developing PDAC, it was of interest to determine if differential expression could be observed within the miRNAs between the two diseases. The PED was used to determine the RNA expression of these MIRs in pancreatitis compared to PDAC. Eight miRNAs (MIR31, MIR96, MIR130B, MIR210, MIR339, MIR429, MIR590, and MIR1208) were downregulated in pancreatitis patients when compared to healthy pancreatic tissue. When the expression of these MIRs were compared between PDAC and pancreatitis, five miRNAs (MIR27A, MIR31, MIR93, MIR130B, and MIR330) were upregulated (Supplementary Table S1).

The final panel of 10 MIRs were chosen systematically using a combination of those which exhibited differential expression between chronic pancreatitis and PDAC as well as other MIRs from our original 18-marker panel. Using the UTSW dataset from cBioPortal, we chose a subset of 10 MIRs (MIR31, MIR93, MIR133A1, MIR210, MIR330, MIR339, MIR425, MIR429, MIR1208, and MIR3620) which, when combined, exhibited alteration across nearly 80% of the PDAC patients (86/109) sampled in the UTSW dataset (Supplementary File S2). Additionally, 5 of these MIRs (MIR31, MIR210, MIR339, MIR429, and MIR1208) exhibited differential expression between chronic pancreatitis and PDAC patients.

The 10 MIRs chosen showed strong potential for utilization as a diagnostic panel as they were altered in a considerable number of patients (86/109) and had clear expression differences between chronic pancreatitis and PDAC. This differential expression provides additional benefits for monitoring high-risk patients. While the expression of these lead MIRs rely on cellular expression data, we predicted that an overexpression in cellular tissue will lead to an abundance of target MIRs being packaged into exosomes, thereby exhibiting an increased expression in PDAC-derived exosomes compared to healthy control samples. These 10 MIRs encompass 18 mature miRs (miR-31-5p, miR-31-3p, miR-93-5p, miR-93-3p, miR-133a-5p, miR-133a-3p, miR-210-5p, miR-210-3p, miR-330-5p, miR-330-3p, miR-339-5p, miR-339-3p, miR-425-5p, miR-425-3p, miR-429, miR-1208, miR-3620-5p, miR-3620-3p) for developing a focused early detection system.

### 3.2. Detection of the miRNA Panel in Pancreatic Cancer In Vitro Models

Cell culture media from each of the cell lines was collected after three days for exosomal RNA isolation, extraction, and analysis by RT-qPCR. It is well established that miRs may not be detectable due to low levels, therefore, any values over 35, or a lack of Cq values were replaced with the arbitrary low value of 36, as previously described [49]. Additionally, calculations were performed as long as 20% of the technical replicates exhibited values less than 35. This method was applied to the data for all four cell lines.

**Figure 2:**
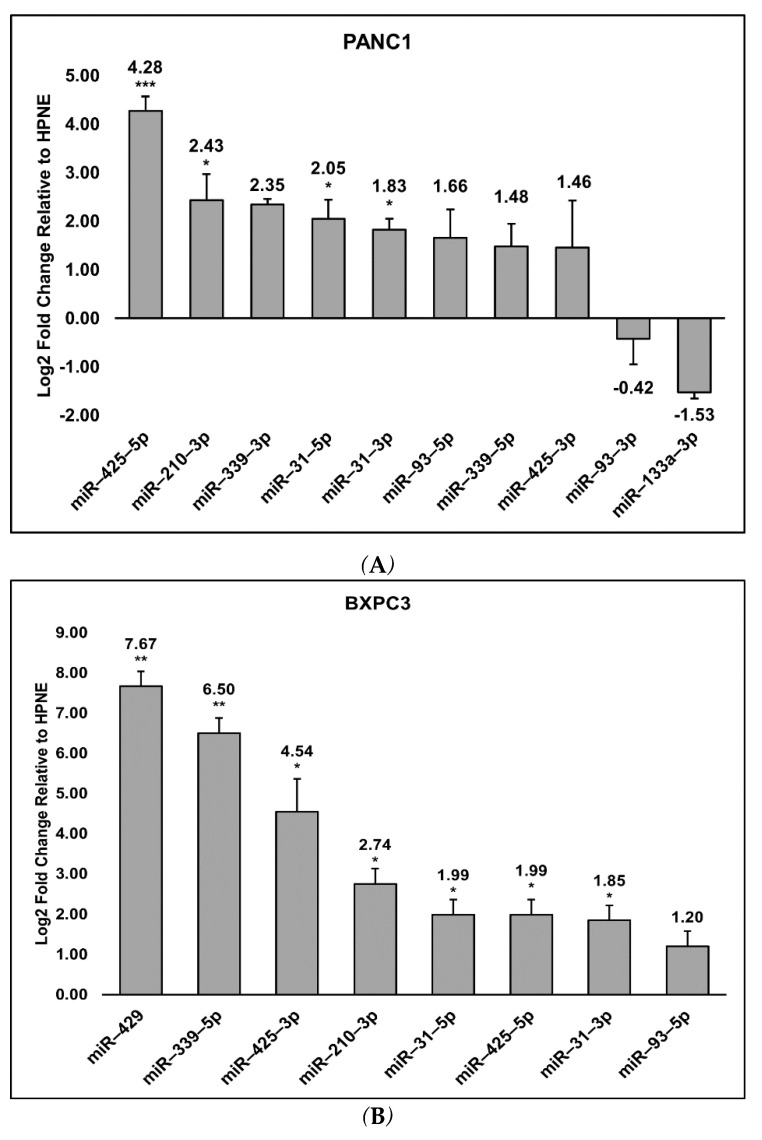

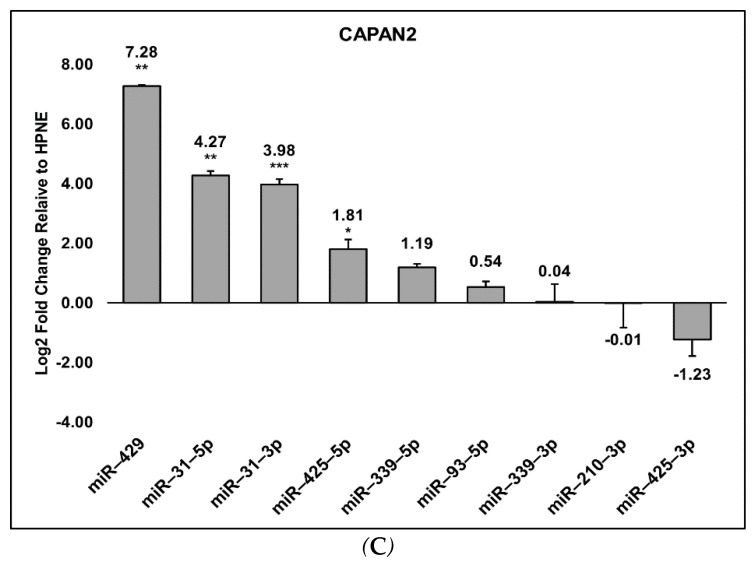
Relative expression levels of MIRs detected in PANC1, BXPC3, and CAPAN2 compared to HPNE. The log2 fold changes of the relative expression levels of miRs in the three different PDAC cell lines, PANC1 (**A**), BXPC3 (**B**), and CAPAN2 (**C**) compared to HPNE was calculated. The log2 fold change was calculated via ΔΔCq values. Significant (*p* ≤ 0.05, *), very significant (*p* ≤ 0.01, **), and extremely significant (*p* ≤ 0.001, ***) are also noted and determined using the student’s *t*-test.

**Figure 3:**
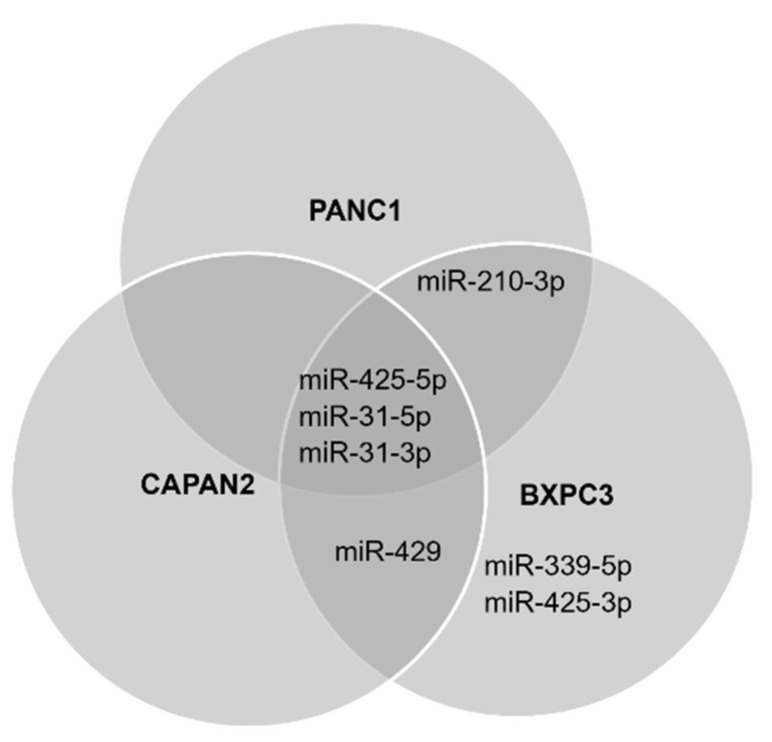
miRNAs differentially expressed in at least one PDAC cell line. The figure exhibits the statistically significant miRs specific to each cell line and which of those were shared. Significant expression of miR-31-5p, miR-31-3p, and miR-425-5p was observed in all three PDAC cell lines. Meanwhile, CAPAN2 and BXPC3 both significantly expressed miR-429. miR-210-3p exhibited significance in the PANC1 and BXPC3 cell culture models while miR-339-5p and miR-425-3p were the only miRs to be significantly expressed in a single cell line, BXPC3.

The expression of the 18 mature miRs was verified using quantitative RT-PCR. All cell lines exhibited log2 fold change expressions relative to HPNE for the following miRs: miR-31-5p (PANC1 and BXPC3 *p* ≤ 0.05; CAPAN2 *p* ≤ 0.001), miR-31-3p (PANC1 *p* ≤ 0.01; BXPC3 *p* ≤ 0.05; CAPAN2 *p* ≤ 0.001), miR-93-5p (PANC1 *p* = 0.0691; BXPC3 *p* = 0.0770; CAPAN2 *p* = 0.2412), miR-210-3p (PANC1 and BXPC3 *p* ≤ 0.05; CAPAN2 *p* = 0.9928), miR-339-5p (PANC1 *p* = 0.1412; BXPC3 *p* ≤ 0.001; CAPAN2 *p* = 0.1499), miR-425-5p (PANC1 *p* ≤ 0.001; BXPC3 *p* ≤ 0.05; CAPAN2 *p* ≤ 0.05), and miR-425-3p (PANC1 *p* = 0.380; BXPC3 *p* ≤ 0.05; CAPAN2 *p* = 0.381) (Figure 2A–C). Only PANC1 exhibited detectable expression changes for miR-93-3p (*p* = 0.7759) and miR-133a-3p (*p* = 0.3000), though neither were significant. miR-339-3p was detected in PANC1 (*p* = 0.1416) and CAPAN2 (*p* = 0.9769), but not in BXPC3. CAPAN2 and BXPC3 also exhibited significant log2 fold change expression for miR-429, *p* ≤ 0.01 (Figure 2B,C). Figure 3 summarizes the miRs which were shared or specific to each cell line.

The data for miR-31-3p and miR-31-5p is consistent with current literature, which has shown a clear link between KRAS mutation and rampant overexpression of miR-31 [51,52,53,54]. Interestingly, overexpression of miR-429 has been observed in PDAC patient-derived xenograft models while also exhibiting associations with decreased metastasis in functional studies [55], increased sensitization to chemotherapy [56], and further suppression of metastasis in in vitro PDAC [57] and hepatocellular carcinoma models [58]. It is unclear why miR-429 may be overexpressed in PDAC models and yet exhibits associations with anti-tumor properties, though it may be due to different underlying biological mechanisms governing in vitro versus in vivo systems, or it could be due to alteration as tumors progress from one stage to the next.

### 3.3. Cell Preferences for Mature miRNA

Each miRNA was analyzed for the presence of both its mature 5p and 3p arms. This was to determine if the cells exhibited preferential expression for an miR to develop a more targeted diagnostic. Previous studies have shown various tumor types exhibit non-equal expression levels for either the 5p or 3p arm of a mature miRNA, whereby one arm is significantly more expressed than the other [59,60,61,62]. The relative expression levels of each arm of the studied miRs were analyzed for significant differences to determine if there was a preference for the 5p or 3p arm in our cell culture models.

**Figure 4:**
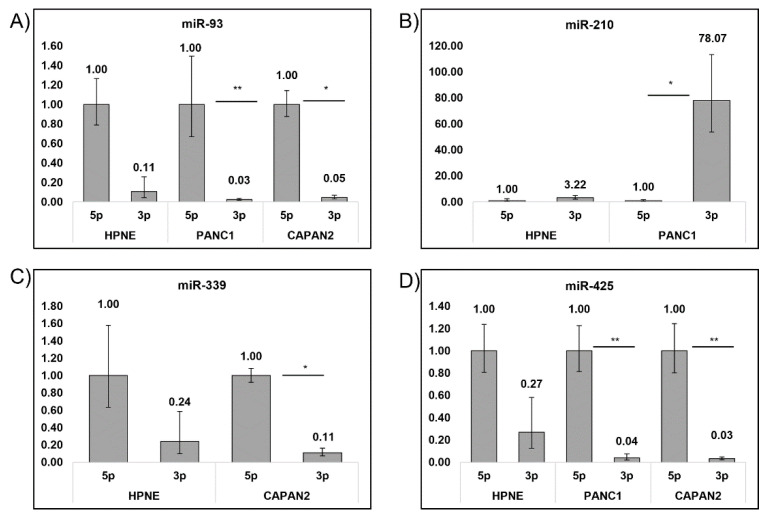
Preference for 5p and 3p arm across PDAC cell lines and HPNE control. Each cell line was examined for a preference in 5p or 3p arm of the mature miRNA Expression levels were calculated relative to the 5p arm of each miR and displayed as fold change values. (**A**) miR-93-5p was preferred in PANC1 and CAPAN2, while (**B**) miR-210-3p was preferred in PANC1. (**C**) miR-339-5p was preferred in CAPAN2 and (**D**) miR-425-5p were preferred in both PANC1 and CAPAN2. Significant (*p* ≤ 0.05, *) and very significant (*p* ≤ 0.01, **) are also noted and were determined using the student’s *t*-test.

There was an overwhelming preference for the mature 5p arm across all cell lines. There was an overwhelming preference for the mature 5p arm across all cell lines. Despite this, only 4 miRs exhibited an appreciable difference between 5p and 3p expression (Figure 4). miR-93-5p reported a statistical significance in expression over the 3p arm in both PANC1 and CAPAN2, *p* ≤ 0.01. The only miR which exhibited a 3p preference was miR-210, in PANC1, and did not exhibit any detectable levels of miR-210-5p (*p* ≤ 0.01). CAPAN2 also exhibited an increase in 5p expression over 3p for miR-339 (*p* ≤ 0.05). Lastly, for both PANC1 and CAPAN2, miR-425-5p significantly overexpressed compared to miR-425-3p (*p* ≤ 0.05 and *p* ≤ 0.01, respectively).

## 4. Discussion

Exosomes have numerous advantages as potential diagnostic vehicles. They provide a snapshot of the internal RNA and protein composition in cells at various stages of disease progression, and their stability in body fluids facilitates the relative ease of collection compared to traditional invasive biopsy methods [63]. Despite numerous ncRNA expression studies in various cancer and tumor systems [37,51,64,65,66,67,68,69,70], the potential clinical application of this breadth of information remains limited and it is only recently that their usage is being examined in several clinical trials [24,71].

In this study, we utilized extensive bioinformatics analysis and enrichment processes to propose a panel of exosomal miRNAs to be used as a potential diagnostic for PDAC. Of the 10 miRs studied, we found seven (miR-31-5p, miR-31-3p, miR-210-3p, miR-339-5p, miR-425-5p, miR-425-3p, and miR-429) that were differentially expressed in PDAC cell lines compared to the control, and also found four (miR-93-5p, miR-210-3p, miR-339-5p, and miR-425-5p) that exhibited a preference for one arm over the other in PDAC cell lines but not arm preference in the control. These biomarkers are strong candidates for the development of a novel non-invasive diagnostic panel for PDAC, with the potential to improve early detection of pancreatic cancer.

Interestingly, miR-133a-5p, miR-210-5p, miR-330-5p, miR-330-3p, miR-1208, miR-3620-5p, and miR-3620-5p were not expressed in any of the tested cell lines. Alternatively, miR-133a-3p was detectable only in the PANC1 cell line. Previous studies suggest that miR-133a, miR-1208, and miR-3620-3p exert tumor suppressive effects [72,73,74,75,76]. The absence of these miRs in the PDAC cell lines may therefore be consistent with the tumor suppression phenotype observed in these previous studies. However, the presence of detectable levels of miR-133a-3p in the PANC1 cell line may be due to the specific mutations associated with this specific cell line. Previous studies have also shown that miR-210-5p is over expressed in bladder cancer, breast cancer, kidney tumors, lung squamous cell carcinoma (LSCC), and stomach cancer but not in PDAC [77]. Meanwhile, miR-210-3p exhibited high levels of expression in bladder, breast, kidney, LSCC, and pancreatic tumors, which is consistent with our observations and further supports tissue-specific expression. Previous studies have found that miR-330-5p may also function as a tumor suppressor by inhibiting PDAC progression, and low levels of the miR have been reported in PDAC tissues [78,79]. Contrary results have been observed with miR-330-3p, with one study suggesting a pro-tumorigenic property in PDAC [80], while others suggesting anti-tumor effects in liver and ovarian cancers [81,82]. Finally, miR-3620-5p is known to form G-quadruplex structures with itself [83]. In the present study, miR-3620-5p was the only miR that had detectable levels of expression (Ct < 35) in the no template controls of the RT-qPCR analysis (data not shown). This was likely due to the miR-3620-5p specific-probe forming a dimer-like structure thus yielding a detectable signal. Thus, it remains difficult to accurately assess miR-3620-5p expression.

In the cBioPortal dataset, MIR31 exhibited deep deletion in 27 patients, which would suggest decreased expression of MIR31 in about 25% of patients. This decrease in expression was not observed in our in vitro models, nor the other 75% of patients sampled in the UTSW dataset. This underscores the variability in expression of MIR between patients and the importance of a multi-marker diagnostic. To that end, our in vitro data for miR-31-5p and miR-31-3p showed significantly upregulated levels in CAPAN2 (miR-31-5p and miR-31-3p) and PANC1 (miR-31-3p) compared to HPNE. This observation is consistent with the literature which has shown a strong positive correlation between mutant KRAS and rampant miR-31 overexpression [51,52,54]. Regardless, it is well established that in vivo and in vitro analyses are often conflicting. For example, one in vitro study utilizing aggressive PDAC cell lines suggests that overexpression of miR-429 correlates with poor survival in later stages [84]. On the other hand, additional in vitro studies state the opposite and have labeled miR-429 as a potential tumor suppressor [56,57,58] as has an in vivo study using patient pancreatic cancer xenografts [55]. Lack of consistency in the expression of target genes amongst the literature is largely due to differences in patient polymorphisms [70,85] as well as potential differences between specific models being used. These variables highlight and support the requirement for diagnostic panels, rather than a single biomarker.

KRAS proto-oncogene and TP53 mutations are ubiquitous for pancreatic cancer. In many cases, a KRAS mutation is the initiating driver of pancreatic tumorigenesis [86]. The KRAS proto-oncogene is a GTPase central to the RAS/MAPK pathway. RAS proteins are crucial to cell growth, proliferation, migration, differentiation, and apoptosis in a tightly regulated cellular system [87]. A single point mutation in residue 12 of glycine to aspartic acid or valine (KRASG12D or KRASG12V) renders KRAS proteins constitutively active [88,89]. This results in rampant growth and proliferation. TP53 is a well-established tumor suppressor, that encodes a cell cycle checkpoint monitor thereby initiating DNA damage/repair pathways and apoptosis in the event that damaged DNA cannot be repaired [90]. A mutation in TP53 results in the inactivation of the protein, allowing for unregulated proliferation when coupled with mutant KRAS. These two hallmark genes of pancreatic cancer serve as a basis for our choice of, PANC1 (p53/KRAS double mutant), CAPAN2 (p53-WT/KRAS-mut), and BXPC3 (p53-mut/KRAS-WT) as robust in vitro models of PDAC. However, there is a possibility that the use of additional in vitro models which exhibit different causative mutations may give rise to differing results than are presented here.

KRAS mutant PANC1 exhibited four statistically significant miRs and CAPAN2 expressed six statistically significant miRs compared to BXPC3, which significantly expressed two miRs. KRAS is well established as the most common mutation in PDAC. Thus, it is possible that the reason why KRAS mutant cell lines yielded a greater number of differentially expressed miRs is due to the ability of constitutively active KRAS to cause rapid growth, via signaling cascades resulting in rapid transcription of genes, thereby enabling higher expression levels of these miRNAs [91,92]. Interestingly, miR-429 exhibited increased expression levels in the KRAS mutant CAPAN2 (Log2 fold change 7.28), and p53 mutant BXPC3 (Log2 fold change 7.67) but was undetectable in the KRAS/p53 double mutant PANC1 model. Previous studies suggest that miR-429 may be a tumor suppressor, despite being commonly overexpressed in PDAC [55]. Given the observed overexpression in CAPAN2 and BXCP3, but not in PANC1, it is possible that p53/KRAS double mutant asserts an antagonistic effect on miR-429, suppressing its expression. Additionally, the overexpression of miR-429 in the KRAS WT BXPC3 as well as KRAS mutant CAPAN2 may predict its beneficial use as a monitor for patients with a mutation in KRAS or P53, but not patients exhibiting mutations in both. Alternatively, this discrepancy may be due to the comparison of PDAC cell lines to an immortalized pancreatic duct model instead of a primary tissue model. The model of HPNE used for this work was originally designed to test the instability of KRAS, thereby providing a model for a precancerous control. Regardless HPNE E6/E7/st is a stable cell line and does not exhibit tumorigenic properties until transfected with mutant KRAS [48]. The HPNE culture we used possesses knockouts of two tumor suppressors (p53 and retinoblastoma, Rb) but maintains a differentiated phenotype and exhibits a precancerous genotype. The use of a precancerous control is a viable option for testing a diagnostic biomarker for early PDAC detection and monitoring.

A preference for the 5p arm of most of the miRs tested was observed in this study. Previous studies have observed this phenomenon with reports of arm switching in tumors [62]. Although arm switching was not observed in this study, the data shows that there was a significant overexpression of the 5p arms compared to the 3p arms in the cancer cell lines compared to the control cell line for MIR93, MIR210, MIR339, andMIR425. This statistically significant overexpression of these mature 5p arms in our miR panel may be beneficial for use as an additional diagnostic feature.

Many of the exosomal miRs identified in this study have also been observed in plasma exosomes and thus may prove beneficial as a diagnostic panel [44,93]. Additionally, six of the 18 MIR transcripts (MIR31, MIR93, MIR210, MIR330, MIR425, and MIR429) identified in the bioinformatics analysis exhibited differential expression between chronic pancreatitis patients and PDAC patients, of which all but MIR93 appear to exhibit overexpression in PDAC models compared to immortalized pancreatic duct model, HPNE. Further work testing the proposed mature miR markers and their expression in healthy, chronic pancreatitis, and PDAC patients is required to determine their efficacy as early markers for monitoring and detection. Additionally, it may be of interest to test this panel in other tumor types in order to establish specificity of the proposed marker. Thus, our proposed diagnostic marker may prove beneficial in identifying PDAC in patients as well as monitoring high risk patients and patients undergoing treatment.

## Figures and Tables

**Table 1:** MIR alterations across pancreatic cancer stages.

Stage I (N = 7)	Stage II (N = 94)	Stage III (N = 6)	Stage IV (IV = 2)
MIR27A	MIR27A		
MIR31	MIR31		MIR27A
MIR93	MIR93	MIR31	
MIR96	MIR96	MIR93	
MIR122	MIR122	MIR96	
MIR130B	MIR130B		
	MIR133A1	MIR130B	
MIR203A	MIR203A	MIR133A1	
MIR210	MIR210		MIR203A
MIR330	MIR330	MIR210	MIR210
MIR339	MIR339	MIR330	
MIR425	MIR425		
MIR429	MIR429		
	MIR522	MIR429	MIR429
	MIR590	MIR522	
	MIR664A	MIR590	
	MIR1208		MIR664A
MIR3620	MIR3620	MIR1208	MIR1208

The cBioPortal tool is a publicly curated database and enables the addition of several different tracks, including a tumor staging track. The tumor stage track was utilized to identify the stages of the 109 patients who participated in the UTSW study. The table reports the number of patients per stage and which MIRs exhibited alteration, defined as either MIR amplification or deletion, for that particular stage. Stage, patient population per stage, and the MIRs altered in each patient sampling are indicated.

## Data Availability

The data that support the findings of this study are available from the corresponding author upon reasonable request.

## Supplementary Materials

The following supporting information can be downloaded at: https://www.mdpi.com/article/10.3390/bios12100831/s1, Supplementary File S1; Supplementary File S2; Supplementary File S3; Supplementary Table S1: MIR expression fingerprint for pancreatitis and pancreatic ductal adenocarcinoma.
